# Supplementary material for: Diminished and Altered Cellular Senescence Response in Delayed Wound Healing of Aging
Source: Aging Cell. 2026 Apr 25;25(5):e70493. doi: 10.1111/acel.70493 (PMC13109648; doi:10.1111/acel.70493)
Supplement: Supplementary file 1 — Table S1: Primer sequences used. [file ACEL-25-e70493-s002.docx]

**Supplementary data**

**Table S1. Primer sequences used.**

| **Gene** | **Forward primer sequence (5’-3’)** | **Reverse primer sequence (5’-3’)** |
| --- | --- | --- |
| p16 | TGTTGAGGCTAGAGAGGATCTTG | CGAATCTGCACCGTAGTTGAGC |
| p21 | CTGAGCGGCCTGAAGATTCC | CCAATCTGCGCTTGGAGTGA |
| p53 | ACGCTTCTCCGAAGACTGG | TCCATGCAGTGAGGTGATG |
| Il-6 | TACCACTTCACAAGTCGGAGGC | CTGCAAGTGCATCATCGTTGTTC |
| MCP-1 | GCTACAAGAGGATCACCAGCAG | GTCTGGACCCATTCCTTCTTGG |
| MMP-3 | CTCTGGAACCTGAGACATCACC | AGGAGTCCTGAGAGATTTGCGC |
| MMP-8 | GATGCTACTACCACACTCCGTG | TAAGCAGCCTGAAGACCGTTGG |
| MMP-9 | ACGACATAGACGGCATCCA | GCTGTGGTTCAGTTGTGGTG |
| TNF-α | GATCGGTCCCCAAAGGGATG | CCACTTGGTGGTTTGTGAGTG |
| TGF-β | ACTGGAGTTGTACGGCAGTG | GGGGCTGATCCCGTTGATTT |
| Col1a1 | CCTCAGGGTATTGCTGGACAAC | CAGAAGGACCTTGTTTGCCAGG |
| Col1a2 | TTCTGTGGGTCCTGCTGGGAAA | TTGTCACCTCGGATGCCTTGAG |
| Col3a1 | GACCAAAAGGTGATGCTGGACAG | CAAGACCTCGTGCTCCAGTTAG |
| β-actin | GCACTGTGTTGGCATAGAGG | GTTCCGATGCCCTGAGGCTCTT |
