# Supplementary figures and images for: Diminished and Altered Cellular Senescence Response in Delayed Wound Healing of Aging
Source: Aging Cell. 2026 Apr 25;25(5):e70493. doi: 10.1111/acel.70493 (PMC13109648; doi:10.1111/acel.70493)

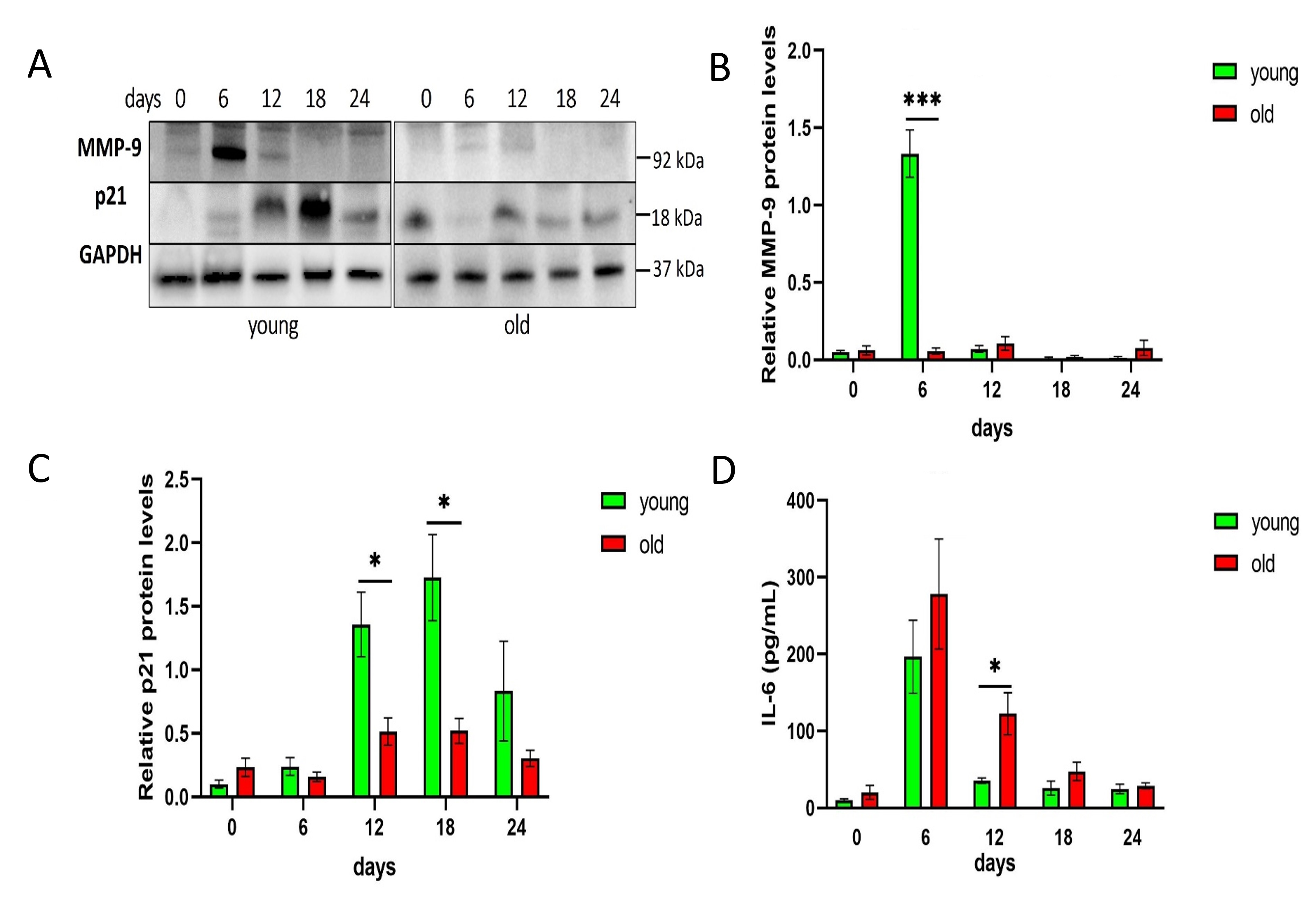

Supplement: Supplementary file 2 — Figure S1: acel70493‐sup‐0002‐Figure‐S1.jpg. Protein expression confirmation of senescence and SASP markers from young and old mouse wounds. Analysis of wound tissue protein lysates from young and aged mice during wound healing. (A) Representative Western blots demonstrating protein levels of MMP‐9 and p21 at 0, 6, 12, 18, and 24 days postwounding. GAPDH is included as a loading control. (B) Quantification of relative MMP‐9 protein levels over time in young and old mice. Protein levels relative to GAPDH from Western blots shown in (A). (C) Quantification of relative p21 protein levels over time in young and old mice. Protein levels relative to GAPDH from Western blots shown in (A). (D) Quantification of IL‐6 protein levels in wound tissue lysates from young and aged mice over the wound healing time course, measured by ELISA. N = 5 per age group per time point. *p < 0.05, ***p < 0.001. [file ACEL-25-e70493-s001.jpg]

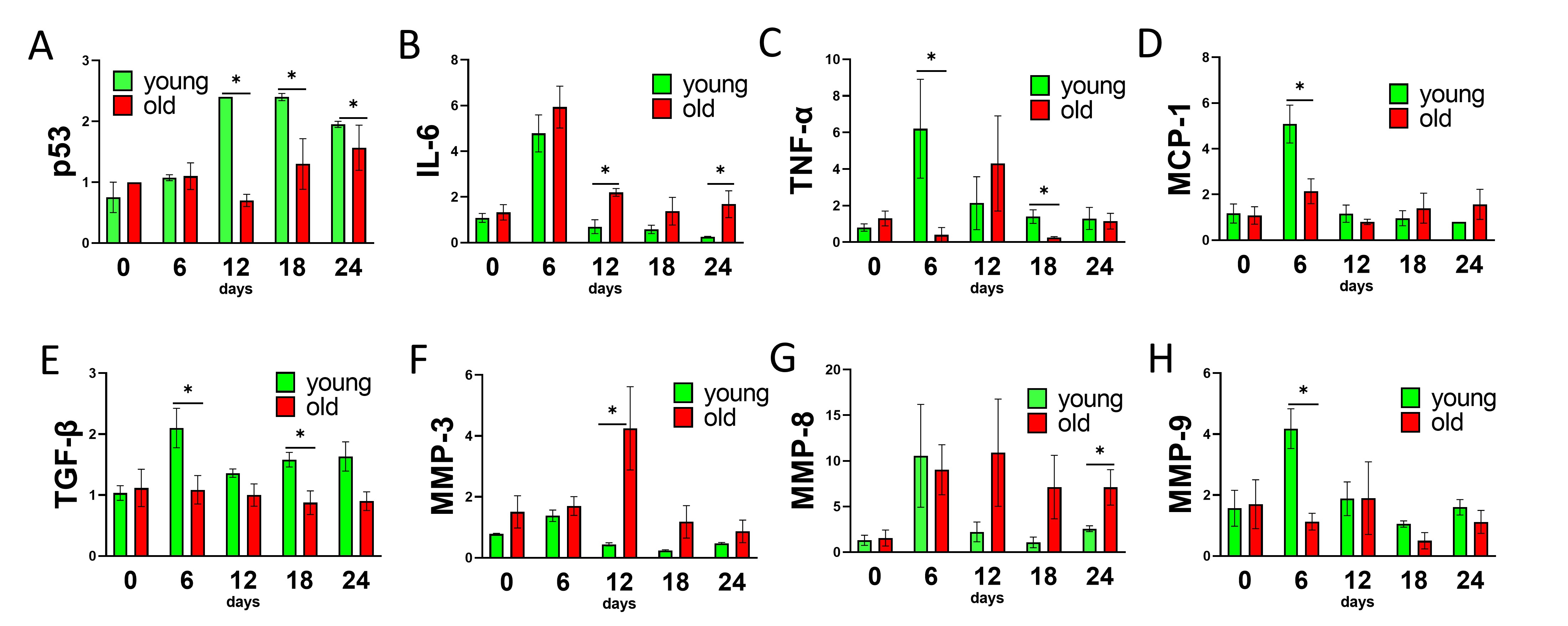

Supplement: Supplementary file 3 — Figure S2: acel70493‐sup‐0003‐Figure‐S2.jpg. Additional senescence and SASP markers during wound healing in young and aged mice. qRT‐PCR analysis of wound tissue from young and aged mice at different time points during wound healing. Expression of (A) p53, (B) Il6, (C) Tnf‐α, (D) Mcp‐1, (E) Tgf‐β, (F) Mmp3, (G) Mmp8, and (H) Mmmp9 is shown relative to β‐actin expression. N = 5 per age group per time point. *p < 0.05. [file ACEL-25-e70493-s007.jpg]

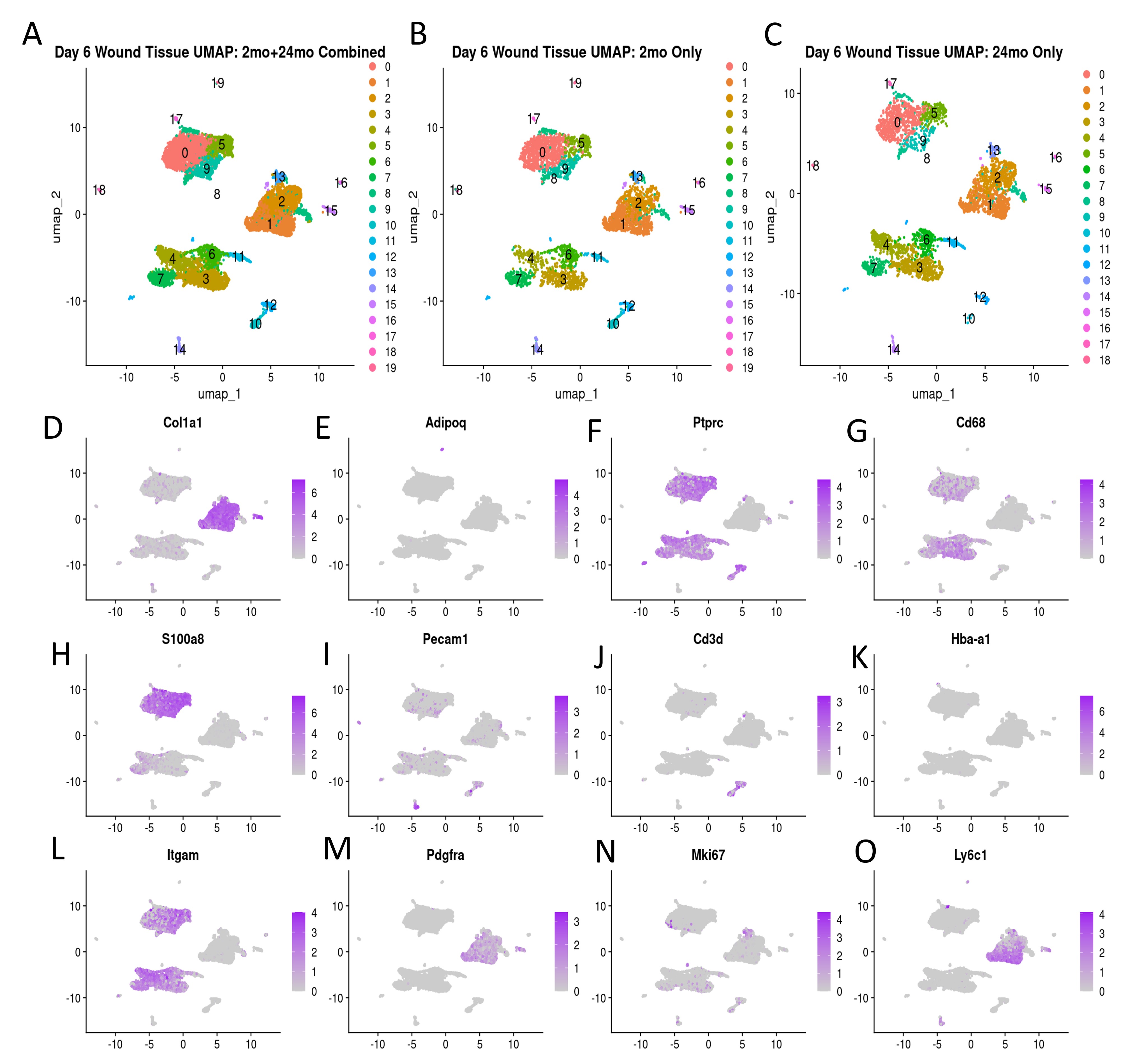

Supplement: Supplementary file 4 — Figure S3: acel70493‐sup‐0004‐Figure‐S3.jpg. Single‐cell RNA sequencing analysis of day 6 wound tissue. (A–C) UMAP plots of single‐cell RNA sequencing data from Day 6 wound tissue. Cells are colored by unsupervised clustering. (A) Combined data from 2‐month and 24‐month‐old mice. (B) Data from 2‐month‐old mice only. (C) Data from 24‐month‐old mice only. Cluster numbers are indicated, and the legend in (A) and (C) demonstrates the corresponding cluster identities based on marker gene expression. (D–O) Feature plots demonstrating the expression levels of selected marker genes overlaid on the combined UMAP from (A). Gene names are indicated above each plot. Color intensity represents the normalized expression level of the gene, with darker shades indicating higher expression. These markers were used to assist in the identification and annotation of cell clusters. (D) Col1a1, (E) Adipoq, (F) Ptprc, (G) Cd68, (H) S100a6, (I) Pecam1, (J) Cd3d, (K) Hba‐a1, (L) Itgam, (M) Pdgfra, (N) Mki67, (O) Ly6c1. [file ACEL-25-e70493-s006.jpg]

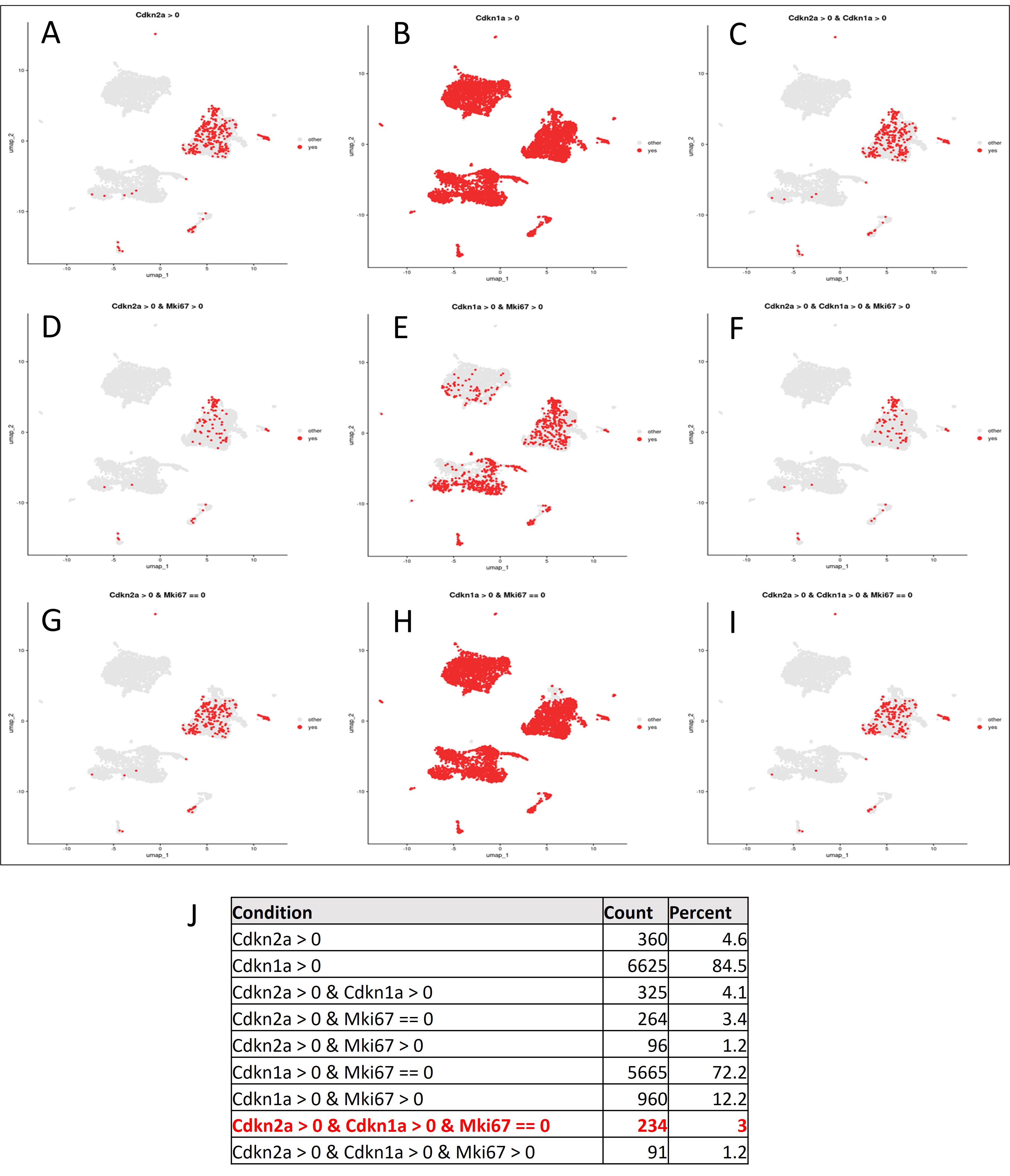

Supplement: Supplementary file 5 — Figure S4: acel70493‐sup‐0005‐Figure‐S4.jpg. Expression analysis of senescence and proliferation markers in wound tissue. (A–I) UMAP plots illustrating the distribution of cells expressing specific senescence and proliferation markers. Cells are colored based on whether they are negative (gray) or positive (red) for the indicated gene or combination of genes. The plots demonstrate cells positive for: (A) Cdkn2a, (B) Cdkn1a, (C) Coexpression of Cdkn2a and Cdkn1a (Cdkn2a > 0 & Cdkn1a > 0), (D) Cdkn2a expression in proliferating cells (Cdkn2a > 0 & Mki67 > 0), (E) Cdkn1a expression in proliferating cells (Cdkn1a > 0 & Mki67 > 0), (F) Coexpression of Cdkn2a and Cdkn1a in proliferating cells (Cdkn2a > 0 & Cdkn1a > 0 & Mki67 > 0), (G) Cdkn2a expression in nonproliferating cells (Cdkn2a > 0 & Mki67 = 0), (H) Cdkn1a expression in nonproliferating cells (Cdkn1a > 0 & Mki67 = 0), (I) Coexpression of Cdkn2a and Cdkn1a in nonproliferating cells (Cdkn2a > 0 & Cdkn1a > 0 & Mki67 = 0). (J) Table summarizing the number and percentage of cells falling into the defined expression categories shown in the UMAP plots. The “Condition” column indicates the expression criteria, “Count” demonstrates the count of cells meeting those criteria, and “Percent” demonstrates the percentage of the total cells analyzed represented by that group. [file ACEL-25-e70493-s003.jpg]

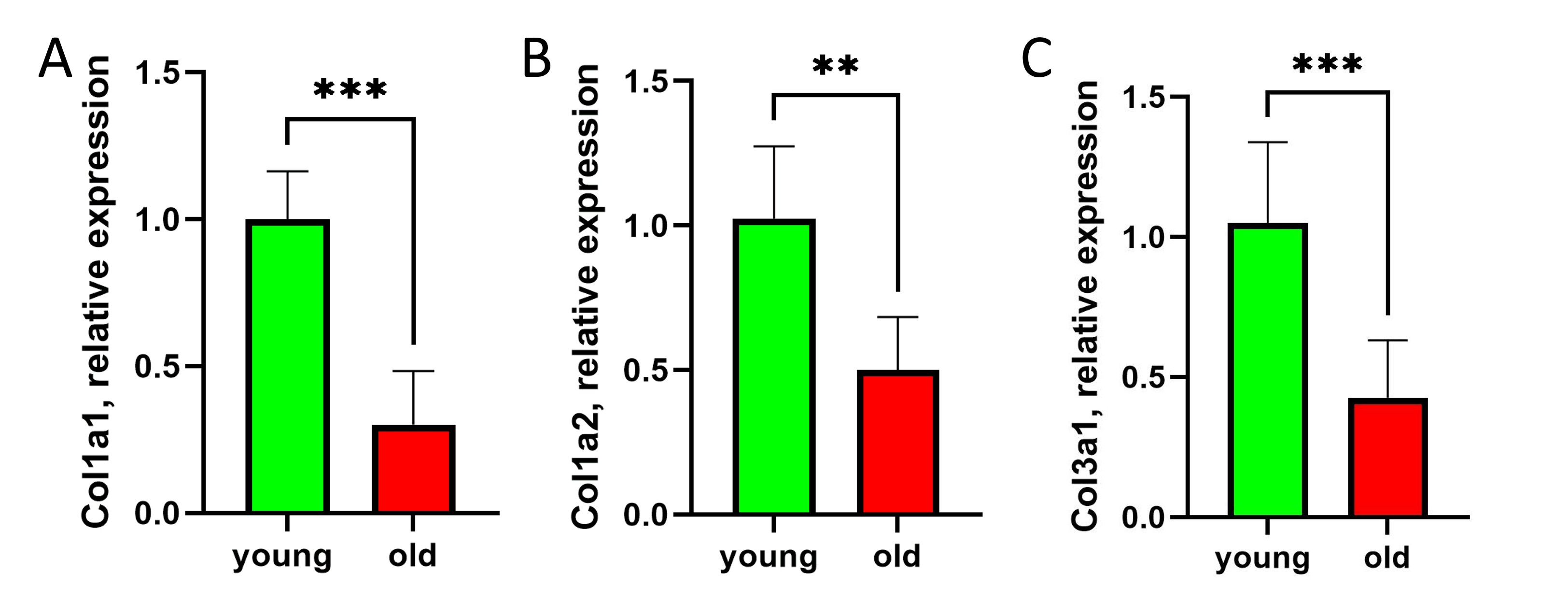

Supplement: Supplementary file 6 — Figure S5: acel70493‐sup‐0006‐Figure‐S5.jpg. Collagen mRNA expression in bulk wound tissue from young and old mice on day 6 during wound healing. qRT‐PCR analysis of wound tissue from young and aged mice on day 6 after wounding. Expression of (A) Col1a1, (B) Col1a2, and (C) Col3a1 are presented relative to β‐actin expression. N = 5 per age group. **p < 0.01, ***p < 0.01. [file ACEL-25-e70493-s005.jpg]

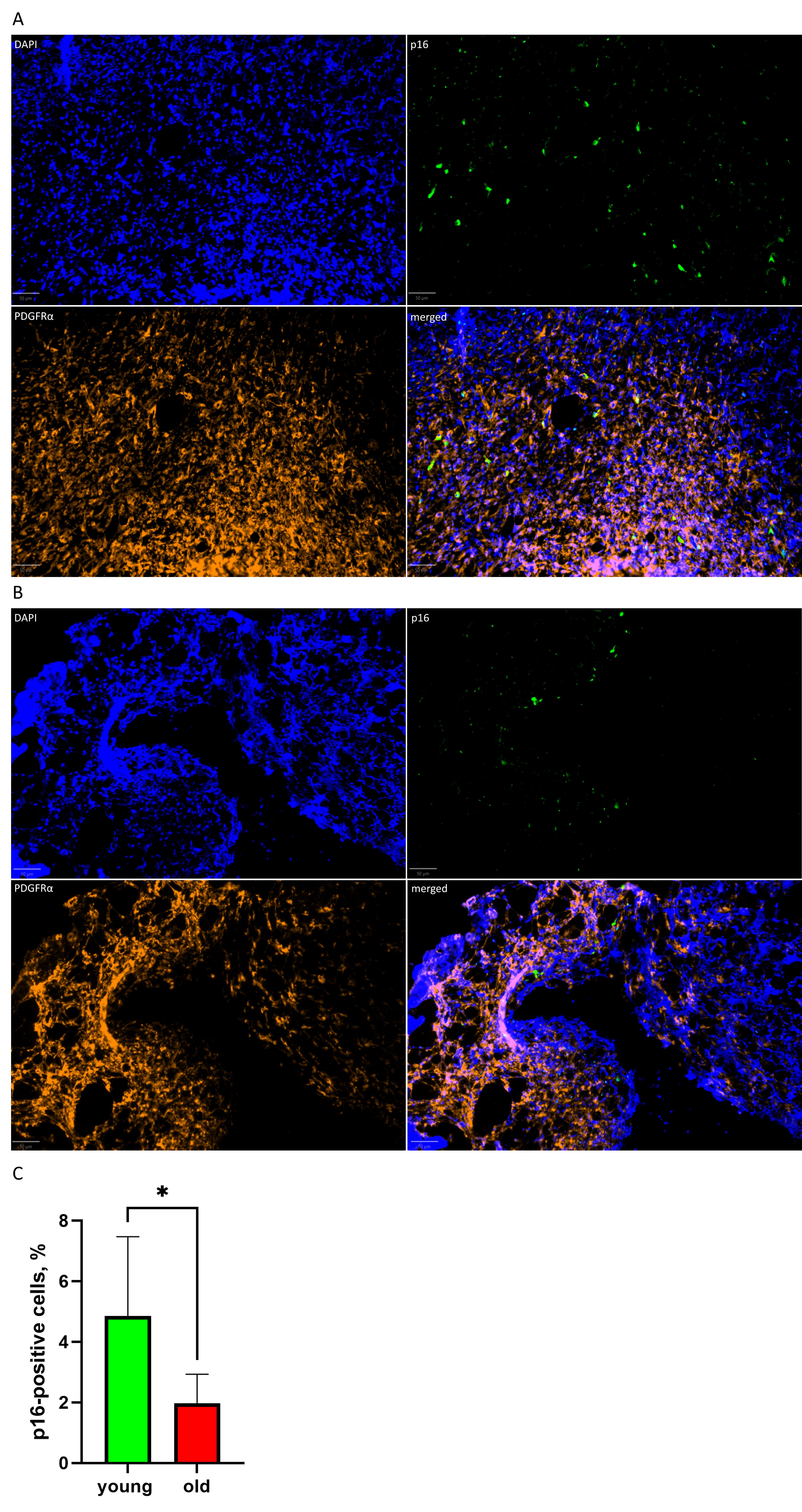

Supplement: Supplementary file 7 — Figure S6: acel70493‐sup‐0007‐Figure‐S6.jpg. p16 is colocalized with Pdgfrα on day 6 after wounding in wound granulation tissue. (A) Representative immunofluorescence images of wound tissue sections from young mice at day 6 postwounding. (B) Representative immunofluorescence images of wound tissue sections from old mice at day 6 postwounding. (C) Quantification of the percentage of p16‐positive cells in the wound tissue of young (green bars) and old (red bars) mice at day 6 postwounding. N = 5 per age group. *p < 0.05. [file ACEL-25-e70493-s004.jpg]
